# Perceived social support and family members of patients with mental disorders: A mixed method study

**DOI:** 10.3389/fpubh.2023.1093282

**Published:** 2023-02-06

**Authors:** Shabnam Amini, Amir Jalali, Rostam Jalali

**Affiliations:** ^1^Department of Psychiatric Nursing, School of Nursing and Midwifery, Kermanshah University of Medical Sciences, Kermanshah, Iran; ^2^Substance Abuse Prevention Research Center, Research Institute for Health, Kermanshah University of Medical Sciences, Kermanshah, Iran; ^3^Department of Nursing, School of Nursing and Midwifery, Kermanshah University of Medical Sciences, Kermanshah, Iran

**Keywords:** perceived social support, mental disorder, family, qualitative study, mixed method study

## Abstract

**Introduction:**

Family members are the caregivers to patients with mental disorders at home, and the mental and spiritual pressures caused by this responsibility necessitate physical, mental, and perceived social support for these families. The present mixed method study is an attempt to elaborate on the perceived social support by Iranian families of patients with chronic mental disorders.

**Materials and methods:**

Using a sequential mixed method explanatory study (quantitative and qualitative), 200 family members of patients with chronic mental disorders were selected through convenient sampling (quantitative phase). Data gathering was started using a demographics form and Stewart and Sherburne's perceived social support scale. The qualitative phase included 10 participants who obtained low scores in the quantitative phase and took part in private, face-to-face, in-depth, and semi-structured interviews. The data obtained in the quantitative phase were analyzed using statistical tests, and the interviews were analyzed using qualitative content analysis.

**Findings:**

The perceived social support by the participants was at a moderate level in different fields of tangible support, information and emotional support, kindness, and positive social interactions. The results of the qualitative phase revealed social support status in 15 subcategories, 6 categories, and 2 themes of support and acceptance by family, relatives, and friends, with support and being understood by society. The results of the qualitative and quantitative sections emphasized the needs of the patient's family members (who acted as caregivers for patients) for support in family, social, and emotional areas.

**Conclusion:**

The family members of patients with chronic mental disorders have different needs in the area of perceived social support. Such needs are more tangible in family members such as children with mentally ill parents or parents with mentally ill children. The results of this study can be used for educational and supportive planning for caregivers of patients, most of whom are family members.

## Introduction

Mental disorders are diagnosable diseases characterized by a defect in cognitive and emotional capabilities. They may emerge when individuals are not able to take care of themselves; however, it is usually the other way around. Therefore, these individuals are not able to undertake their daily activities (1). About 27% of the adults living in European countries are found to be suffering from at least one mental disorder over the past 12 months. These mental disorders represent about 13% of the total disease load, and the projection of this figure for 2020 is 16% (2). The disease creates functional limitations for patients in different areas of life and this makes it essential to provide care to these patients (3).

Studies in Asian countries have shown that about 70% of patients with chronic mental disorders live with their relatives and family members, who are in charge of taking care of these patients (4). The families' responses and functions with regard to the presence of a mentally ill patient and the pressures are not the same (5). Looking after patients with chronic mental disorders creates emotional burnout and might have destructive mental and spiritual effects on the caregivers (6). Family members play a key role in providing care and treatment to members with mental disorders (7). Therefore, the load of responsibility of looking after mentally ill patients is mostly on the family members (8). This situation and the consequences might have notable effects on the daily lives of caregivers and impose a risk to their mental and physical health (9). Providing continuous care for mentally ill patients isolates the caregivers and makes them more vulnerable to social hardships and economic crises (10). Therefore, it is essential to provide proper perceived social support to preserve and improve the physical and mental health of these individuals (11). Perceived social support is the social support that is available to a person when needed (12).

Proper social support for the families of patients who are mentally ill helps them to accept the patient's condition and also giving emphasis on the spiritual dimension and religious beliefs can have a significant impact on the physical and mental condition of the family caregivers (13). Family caregivers of patients with mental disorders are more affected compared to caregivers of patients with other diseases and need more support (14, 15). Financial limitations, lack of appropriate perceived social support, family dysfunction, stigma, discrimination, and the destructive behavior of the patient are the main problems of these families (13). In Iran, failure to meet the needs of caregivers, job burnout, a high burden of caregiving, high social stigma, low social support for caregivers, and low quality of life for caregivers are among the most important challenges faced by caregivers. These factors greatly affect caregivers' quality of life (16).

Wong et al. reported that emotional support was one of the social supports with the highest effect on the psychophysical health of caregivers (17). Muñoz-Bermejo et al. argued that perceived social support was the stronger source of support for family caregivers (18). The provision of quality services to meet the needs of families can improve the quality of lives of caregivers and attenuate the pressure on family systems (19). Families' needs for support, depending on family condition, include the development and growth of family members and coordination and interaction among family members (20). Therefore, interaction with families is one of the ways to find out about psychosocial needs, and through a better perception of such needs, better solutions can be planned (21). Therefore, one of the best ways to learn about perceived social support in families is to hear and reflect on their opinions (20).

Using different research paradigms is an efficient and useful approach despite the complicated nature of such systems and research environments, a promising study cannot merely rely on a qualitative or quantitative method to achieve a relatively adequate knowledge of a specific situation. To learn about the nature of a social problem and its solution, a mixture of qualitative and quantitative methods is needed (22). Using quantitative and qualitative approaches yields a better understanding of the research question compared to using these approaches separately (23). Mixed research methods (24) can examine perceived social support in families through quantitative methods using standard questionnaires and at the same time yield deep insights through a qualitative examination of statements and the opinion of subjects in a natural environment. Therefore, the present study is an attempt to elaborate on perceived social support by families of patients with chronic mental disorders.

## Materials and methods

### Design

The study was carried out using the sequential mixed method explanatory study (25) including a cross-sectional study in the quantitative phase and content analysis in the qualitative phase to find a more realistic and tangible image of social support.

### Setting

The quantitative phase was done with the participation of 200 family members of patients with chronic mental disorders at home who visited the Farabi and Mohammad Kermanshahi centers in Kermanshah, Iran.

Farabi hospital is an educational, specialized, and subspecialty center for the mentally ill that provides educational, medical, and research services. This hospital has 250 active patient beds and different departments related to the diagnosis, treatment, and rehabilitation of psychological disorders and provides medical services for admitted adult patients and outpatients. Mohammad Kermanshahi Hospital is also an educational, medical, and research center, specialized and sub-specialized for children and adolescents. This center has a special section for children and adolescents (30 active patient beds) with mental disorders and provides all diagnostic, treatment, and rehabilitation services for these patients as outpatient and inpatient services. Both centers are teaching hospitals that accept patients from cities and provinces nearby.

### Participants

The participants were selected through the convenient sampling method based on inclusion criteria (viz., desire to participate, at least one chronic mental patient at home, age range 18–60 years, and a history of living with patients with chronic mental disorders for at least 6 months). Inclusion criteria were assessed based on interviews with participants. The participants were all the families of patients with chronic mental disorders referred to Farabi and Mohammad Kermanshahi hospitals in Kermanshah Province. In a quantitative study, from April 2019 to November 2019, the researcher visited the mentioned hospitals during the morning and evening shifts and selected the participants based on the criteria and through the convenience sampling method. Because of the small number of patients admitted to Mohammad Kermanshahi hospital, only 13% of the samples were from this hospital, and the rest of the samples were selected from Farabi hospital, Kermanshah.

The required sample size was calculated using the sample size formula. Using previous studies (26), 95% confidence, and 10% accuracy, the sample size was equal to 346 people. Given the number of patients hospitalized in Kermanshah and the inclusion criteria, only 200 people agreed to participate in the study for 6 months.

The qualitative phase was carried out by selecting the participant through a purposeful method from among the participants in the quantitative phase and based on their social support score, and those with lower social support scores were selected. Interest in participation, permission to record conversations, and the ability to speak Farsi and the local dialects were among the criteria for entering the qualitative section. In the qualitative sampling, the research team tried to select the informant persons who had extensive information with a social support score in the quantitative part of less than 40%, while observing maximum variation in sampling. Sampling was continued until data saturation (10 participants as shown in Table 1).

**Table 1:** Characters of participants.

**No**.	**Age year**	**Marital status**	**Educational level**	**Relative to the client**	**Job**	**Social support scale score**
1	59	Widowed	Elementary level	Mother	Manual worker	31
2	40	Unmarried	Secondary level	Sister	Housewife	19
3	18	Unmarried	High school student	Son	Student	38
4	24	Unmarried	High school diploma	Brother	Manual worker	28
5	54	Married	High school diploma	Father	Shopkeeper	31
6	30	Unmarried	University level	Sister	Manual worker	24
7	19	Unmarried	High school diploma	Daughter	Student	19
8	60	Widowed	Elementary level	Mother	Housewife	29
9	46	Married	High school diploma	Spouse	Employee	36
10	46	Married	University level	Spouse	Employee	25

### Data collection

After selecting the participants in the quantitative phase and securing their consent to participate, a demographics form and Stewart and Sherbourne support scale were used for data collection. In order to collect data, after selecting the participants through convenient sampling, one or two family members (preferably parents and children) were selected for each of the clients with mental disorders. Then, a research colleague administered demographic and social support questionnaires after briefing them and answering their questions if any. Data collection was done from August to the end of December 2019.

The interviews of the qualitative phase were private and face-to-face on different occasions (morning and evening) at the participants' convenience and in Farabi and Mohammad Kermanshahi hospitals (by the first author). Extra data such as voice tone, body movement, cry, laughter, and shaky voice were also recorded. The interview duration varied depending on the energy of the participants. The average time of interviews was 50–60 min, and in some cases, the interviews took about 80 min. In addition, all interviews were conducted in the native language, and the interviewer was familiar with the local accent and had passed a qualitative research workshop, which helped him to glean richer information. All stages of the interviews were supervised by the research team (second and third authors) who had sufficient familiarity and mastery of the interview and qualitative research. All participants participated in one interview session.

The interviews would be continued based on the guiding questions, of which experts had confirmed the content validity beforehand. The guiding questions were designed by the research team.

Some of the questions asked in the interviews are as follows:

What are the effects of having a family member with a chronic mental disorder on social and professional relationships?

As a member of the family with a chronic mental patient, what are the supports you expect from relatives, friends, and society?

What measures by families with chronic mental patients lead to receiving more social support?

Open questions would be asked during the interviews based on the participants' responses to shed more light on the details. By directing and using interview techniques such as active listening and guiding, the interviewer helped the participants to find the main concepts. As the study went on, the interviewer modified the questions based on the key categories. In other words, gradual data analysis and the extraction of categories dictated the path of the next interviews. At the end of each interview, the participant would be asked “Is there anything you want to talk about? And do you have any question?” After the 10th interview, data saturation was realized and the interviews were ended.

### Sherbourne and Stewart social support scale

With 19 statements and four subscales, the scale measures perceived social support by the respondent. The subscales are tangible support (statements 9–12), information and emotional support (statements 1–8), kindness (statements 16–18), positive social interaction (statements 13–15), and the last statement (19) is an extra statement. It is a self-report tool designed based on Likert's 5-point scale (never = 1, rarely = 2, sometimes = 3, most of the time = 4, and always = 5). The lowest score on this test is 19, and the highest score is 95. According to the designers, reliability coefficients based on Cronbach's alpha for the subscales emotional support, information support, tangible support, positive interaction, kindness, and the whole scale are 0.96, 0.96, 0.92, 0.94, 0.91, and 0.97, respectively (27). Face and content validities are supported by psychologists, and according to (28), the reliability of the scale is 0.97.

### Data analysis

Descriptive and inferential statistics were used in the quantitative phase using SPSS (v.25). In this section, Kolmogorov–Smirnov, chi-square, Mann–Whitney U, and Kruskal–Wallis tests were used, and all steps were performed in SPSS software version 25. The qualitative phase was carried out based on the Graneheim and Lundman qualitative content analysis method (29) for data analysis; that is, each interview would be followed by listening to the recorded voice, and then the recorded voice would be listened to for the second time and line-by-line, so that each line could be transcribed before listening to the next line. Afterward, the researcher would read all the interviews to develop a general perception of the content. Based on this perception, semantic units, primary codes, the primary categorization of the codes, general categories, hidden content in the data, and themes were extracted. The first author performed data collection and analysis under the supervision of the second and third authors. The second and third authors had passed courses on qualitative research, conducting such studies with sufficient mastery in the qualitative analysis of data in various fields, and the publication of various quantitative and qualitative articles. The data analysis steps were performed in the Persian language and manually without using the software.

### Rigor

To ensure data reliability and validity, the four measures of dependability, confirmability, credibility, and transferability (30) were used. To increase credibility, researchers engaged with the participants and the research field for a long period of time continuously. The researchers ensured the widest diversity in terms of age, gender, education, and kinship relation with participants. In addition, adequate time was spent on data gathering, and the researchers had a long-term engagement with the data. Continuous mental engagement with data increased the breadth and depth of information. The results of the data analysis were given to the participants for confirmation (member check). Conformability was achieved by discarding the researcher's prejudices and ideas and observing the principle of neutrality in collecting, analyzing, and disseminating findings. In addition, the opinions of experts familiar with the subject were used. The codes, data, and a summary of the transcriptions were provided to the experts for extracting meaning and receiving feedback for the final report. To control the dependability and stability of the findings, the interviews were implemented and coded as soon as possible by two members of the research team separately. The findings were then shared with other collaborating and non-collaborating researchers and their corrective comments were applied.

To achieve transferability, a rich description was written with details about the environment and the participants. In addition, the demographic characteristics of the participants were mentioned and many direct quotes were used.

## Results

### Results of the quantitative phase

The results of the research in quantitative part showed that 51% of the participants were married, 65.5% were residents of the city, and 25.5% were employees. According to the findings, 39% of the research units had completed a university education. Moreover, 28.5% of the participants were children whose parents had mental disorders (Table 2). The average age of research subjects was 37.04 ± 11.67 years, and the average monthly income was about $698.56 ± 25.3.

**Table 2:** Demographic variables of research units in quantitative study section and perceived social support condition.

**Variables**		***N* (%)**	**Perceived social support**	**z**	***P* value**
			**Mean ±SD**		
Marital status	Married	102 (51)	62.19 ± 18.58	0.059	0.97
	Single	81 (40.5)	62.26 ± 21.34		
	Divorced widow	17 (8.5)	60.65 ± 20.17		
Education	High school	48 (24)	61.14 ± 19.39	0.26	0.88
	High-school diploma	74 (37)	62.8 ± 21.13		
	Higher Edu.	78 (39)	62 ± 18.91		
Employment status	Housewife	44 (22)	58.327 ± 18.99	1.22	0.75
	Manual worker	35 (17.5)	61.94 ± 19.09		
	Employee	52 (26)	63.3 ± 20.92		
	Unemployed	40 (20)	60.55 ± 22.75		
Self employed	29 (14.5)	68.76 ± 15.32		
Location	Urban	131 (65.5)	62.4 ± 19.17	−0.098	0.92
	Rural	69 (34.5)	61.49 ± 21.03		
Relative to the patient	Spouse	20 (10)	60.75 ± 20.07	9.84	0.04
	Brother	50 (25)	63.56 ± 19.11		
	Sister	42 (21)	66.48 ± 20.64		
	Child	57 (28.5)	55.91 ± 18.91		
	Parent	31 (15.5)	62.19 ± 18.58		

According to the minimum and maximum score of each subscale, the results that showed the average score of the five subscales were at moderate to desirable levels (Table 3). The chi-squared, Mann–Whitney, and Kruskal–Wallis tests showed no significant difference in terms of tangible social support based on demographical variables (*P* > 0.05). There was only a significant difference between occupation and kinship relation with patients in terms of information and emotional support subscales (*P* < 0.05). In general, there was a significant relationship between social support and kinship with patients (*P* < 0.05). The Spearman correlation test showed a direct and insignificant relationship between age and social support (*P* > 0.05). In addition, the results of the Spearman correlation test showed an inverse and insignificant relationship between monthly income and social support and the subscales (*P* > 0.05).

**Table 3:** Perceived social support and its sub-scale in participants in study.

**Variable category**	**Min**	**Max**	**Mean (sd)**
Social support	19	95	62.09 (19.78)
Tangible support	4	20	13.12 (4.3)
Information/emotional support	8	40	25.7 (8.5)
Affectionate support	3	15	10.06 (3.49)
Positive social interaction	3	15	9.86 (3.53)

The results of the Spearman correlation coefficient test showed that there was a direct relationship between age and social support, which was not significant at a 95% confidence level (*P* < 0.05). Moreover, age had an inverse and insignificant relationship with the tangible support subscale (*P* < 0.05) and a direct and insignificant relationship with the other subscales (*P* < 0.05).

Moreover, the results of the Spearman correlation coefficient test showed that there was an inverse and insignificant relationship between the average monthly household income and social support and its subscales at a 95% confidence level (*P* < 0.05) (Table 4).

**Table 4:** Relationship between social support and its subscales with demographic variables of research units in quantitative study section.

**Variables**		**Social support**		**Sub-scales of social support**				
		**Mean** ±**SD**	***P***	**Tangible support**	**Information support**	**Emotional support**	**Affectionate support**	**Positive social interaction**
Marital status^*^	Married	62.19 ± 18.58	0.97	0.69	0.99	0.99	0.99	0.86
	Single	62.26 ± 21.34						
	Divorced and widow	60.65 ± 20.17						
Education^*^	High school	61.14 ± 19.39	0.88	0.74	0.8	0.8	0.95	0.94
	High-school diploma	62.8 ± 21.13						
	Higher Edu.	62 ± 18.91						
Employment status^*^	housewife	58.327 ± 18.99	0.75	0.56	0.7	0.7	0.63	0.75
	manual worker	61.94 ± 19.09						
	Employee	63.3 ± 20.92						
	Unemployed	60.55 ± 22.75						
	self-employed	68.76 ± 15.32						
Location^**^	Urban	62.4 ± 19.17	0.92	0.18	0.012	0.012	0.17	0.084
	Rural	61.49 ± 21.03						
Relationship to the patient^*^	Spouse	60.75 ± 20.07	0.04	0.82	0.85	0.85	0.7	0.79
	Brother	63.56 ± 19.11						
	sister	66.48 ± 20.64						
	Child	55.91 ± 18.91						
	Parent	62.19 ± 18.58						

^*^Kruskal Wallis Test.

^**^U Mann- Whitney Test.

### Results of the qualitative phase

A total of 381 codes, 15 subcategories, 6 categories, and 2 themes (support and acceptance by family, relatives, and friends; support and understanding by society) were found. The categories and subcategories obtained from the analysis of qualitative data are given in the quantitative section in Table 5.

**Table 5:** Lists the categories and subcategories in qualitative phase versus of sub-scales-of social support in quantitative phase.

**Sub-scales-of social support**	**Subcategories**	**Categories**	**Theme**
Tangible support	Financial	Need for supporter	Support and acceptance by family, relatives
Emotional support	Emotional		
Affectionate Support	Spiritual		
Affectionate Support	Need for attention,	Concerns about being accepted	
Emotional support	Empathy		
Positive social interaction	Acceptance		
Emotional support	Positive attitude	Not being judged	
Positive social interaction	Not being deserted		
Tangible support	Welfare concerns	Social support concerns	Friends; support and understanding by society
Information support	Health concerns		
Positive social interaction	Interactive concern		
Positive social interaction	Negligence of citizenship rights	Concerns about social acceptance	
Positive social interaction	Isolation, and stigmatization		
Emotional support	Unpleasant experience	Uncertain future	
Tangible and emotional support	Distress		

#### Support and acceptance by family, relatives, and friends

The theme consists of three categories and eight subcategories. All the participants mentioned the need for a financial and emotional supporter, as well as the need to not be judged and to be accepted.

##### Need for supporter

Analyses showed that the availability or lack of financial, emotional, and spiritual support to the family members, where parents or children are caregivers in particular, was a key factor in the sustainability of careers; that is, when the family has no financial concerns, they can concentrate more on the treatment. Some participants expressed those as follows:

“*Since my mother's disease has become worse, my father started to say that spending money on crazies is a waste of money…” (P3)*.

“*I always wanted to have my mother's attention, but she is too busy taking care of my father. She would never see me and everyone had forgotten me…” (P7)*.

“*As far as I remember, my parents were too busy taking care of my sister to pay attention to me. My sister had schizophrenia and she had all the attention at home. I wished she would die sooner so that I could receive a bit of attention from my parents” (P6)*.

##### Concerns about being accepted

One of the main factors in social support for families of patients with chronic mental disorders was concerns about being accepted. The participants' comments showed that the need for attention, empathy, and acceptance were the main factors in creating the sense of being accepted, which made the families more persistent in taking care of their patients. Some of the participants expressed those as follows:

“*Since people have found out that my father is hospitalized due to mental problems, nobody takes me seriously. There is no respect and acceptance for me and I am totally invisible…” (P7)*.

“*I need someone to talk to when I feel sad or down, but there is none…” (P1)*.

“*I would like to find other families who have mental patients like us. We can be good* friends *for each other as we can better understand each other…” (P6)*.

##### Not being judged

Interviews analyses showed that being safe from others' judgment was a key factor in family members' ability to accept and cope with their situation. This category consisted of two subcategories such as a positive attitude and not being deserted. Some of the comments are as follows:

“*I worry about the time that my mother is discharged from the hospital and people in the neighborhood would start calling her crazy…” (P10)*.

“*After finding out about my sister's mental problem, my husband wants us to break up. He does not want to live with me, he says that when I grow old, I will develop mental disorders as well…” (P2)*.

“*Back in the hospital, they gave papers and colored pencils to my mother to draw paintings as a treatment. Our relatives made a laughing stock of her for her childish paintings when she was at home so that she returned to hospital in two days…” (P3)*.

#### Concerns about support and being understood by society

The concept of support and being understood in society was revealed in the analyses of interviews. This theme is comprised of three categories and seven subcategories. Qualitative analysis of the interviews showed that the participants in the study had several problems in this regard.

##### Social support concerns

Social support concerns in areas such as welfare, health-related programs, and social interactions were the main issues that were frequently mentioned by the participants. This category is comprised of three subcategories, namely, welfare concerns, health concerns, and interactive concerns.

“*I wish there was at least financial support for these patients so that we would not have financial concerns for hospitalization costs or borrow money from friends and relatives to pay the hospital or medicines bills…” (P4)*.

“*I wish I was treated like a normal person and there were no whispers or pointing in the neighborhood when my father and I went outside…” (P7)*.

##### Concerns about social acceptance

The interview analyses revealed that the cases of negligence of citizenship rights, isolation, and stigmatization as major factors in mental and emotional problems that led to social isolation.

“*Kids at school make sure that every new friend that I find knows that my father is hospitalized. They cut their friendship immediately as if I have HIV…” (P9)*.

“*When my daughter was engaged, my daughter in law told the bridegroom family ‘welcome to the family of chained and always hospitalized crazies…” (P1)*.

“*I am always worried that my friends might see me when I go to hospital to visit my father. I always look for excuses to cancel the visit; but I love him and cannot convince myself not to pay a visit…” (P7)*.

##### Uncertain future

The majority of the families had turbulent and stressful life and were concerned about their uncertain future in terms of social security. There were two subcategories in this category including unpleasant experience and distress.

“*Since the last time that her family rejected my marriage proposal because of my mother's disease, I feel no energy in life and wish for death. My life is wasted and I see no bright future ahead…” (P3)*.

“*I check everything to make sure that my children have no mental diseases. They say that* mental *disease can be inherited from grandparents and parents…” (P9)*.

## Discussion

The results showed that understanding and showing empathy to family members and relatives were the most important request of the participants in terms of family and relatives' support and acceptance. According to studies, family members' understanding of interactions and support between oneself and friends, relatives, and society, in particular, can be a key factor in the interaction with patients and the treatment process (16, 31). When empathy and unity are strong among family members, relatives, and friends, the family can be more responsible toward the patient and provide better support and therapeutic and rehabilitation solutions for the patient (20). Ebrahim et al. argued that the presence of a disabled individual in the family is a strong stressor and disrupts the balance in the family system (32). To elaborate on the necessity of perceived social support from family members' viewpoint, it is notable that family members of a patient with a mental disorder experience mental pressures and become more vulnerable and sensitive due to the mental pressure, severe physical and emotional fatigue throughout the treatment process, and the costs of medication and providing care to the patient (33). In addition, care pressure on the family members, as caregivers, creates physical and mental burnout over time (34).

Quantitative results showed that the kindness level was at a moderate-desirable level, and the qualitative results specifically emphasized kindness. However, the results frequently highlighted emotional needs along with empathy. As the results showed, the parents of children with mental disorders required extensive emotional support in different areas given the mental pressures on them. Derguy et al. reported the emotional needs of the parents with autism, as a mental disorder, and noted that the need for emotional support was one of the main aspects of mental needs in these parents (35). To elaborate on the findings, emotional support can help these families with the treatment and rehabilitation process. Studies have shown that expression of emotions can be a psychological response by the caregivers and other family members toward the abnormal behavior of the patient with a mental disorder (36).

Emotional and informational supports were at a moderate level, and the extent of support in this area had a significant relationship with kinship relation between caregivers and patients; that is, social support in terms of emotional and informational support in the children with mentally ill parents was at a low level. Qualitative studies have emphasized emotional support mainly in the area related to mental problems. The care-seekers needed support in areas like not being judged, not being socially deserted, and no social stigmatization. Lack of these supports triggers mental problems and behavioral changes in particular. Studies have shown that families of patients with mental disorders experience heavy pressure in life (9, 14). Emotional support for family members during crises (37) and the mental disorder of one of the members functioned as a proper protective factor to improve the mental health of the rest of the family members and lower mental pressure (15).

Emotional support and information about illness, how to care for it, and how to use the support and social resources available were the important quantitative outcomes. The qualitative findings also highlighted concerns about support by society, consultation services, provision of information, concerns about the future, and the fear of an uncertain future. These concerns were rooted in a lack of information about the disease and prognosis, which is consistent with the quantitative findings. Many families mentioned a lack of information about mental illnesses as a key factor in the way their relatives treated them and their interactions with friends and relatives. Ong et al. concluded that the need for informational support was one of the main needs in the families of patients with mental disorders (38). The informational needs of about 30% of the families were not met properly, while the information needs of the rest of the families were met at a moderate or good level. The number of participants in the study and the study tools can affect the findings.

Quantitative findings revealed that positive social interaction was a key factor that was affected by many other factors. The qualitative results showed that one of these factors was the fear of improper responses by friends, relatives, and society. The needs for social relationships and social acceptance were the subcategories of social support concern, which is consistent with quantitative results. Studies have shown that one of the most common and most challenging social pressures on these families is stigma (32, 39, 40). The stigmatizations are mostly due to the general perspective of the community about the lack of competence of the family to look after the patient has led to the initiation or intensification of the disease (32). The blame is usually followed by a sense of shame in the family, and to adapt to it, the family tries to avoid social situations and keep the patient hidden from society (40). Karanci et al. argued that improper intervention of the patient and family, deserting, and belittling might create negative perceptions in family members and even eliminate their ability to care for the patient (11). Clearly, the majority of the studies in this field have mentioned that the sense of being belittled by others and society was the key factor in the fear of being deserted in these families. There is a need for more studies and better programming in this area.

Another need mentioned by the qualitative results was welfare concerns. The hardships of treatment and the disease and providing care to a family member create several financial problems for the family. Many of the participants complained about the heavy costs of treatment and their inability to keep their normal job due to the problems of the disease. Iseselo et al. showed that the families of clients with mental disorders face a wide range of problems and needs. One of the main needs is the need for financial and social support (13). Bashir et al. believed that the majority of the parents were under heavy pressure due to the expenses of medical care for their children (41). Hartley and Schultz showed that financial needs and interruption of family revenue were the main factors in the need for social support (42). Falk et al. maintained that the parents' needs for economic and financial support were one of the effective factors in the mental health of parents of a child with a mental disorder (43).

The concern about being accepted, uncertain future, and distress and worries among family members were common problems. The participants emphasized the sense of security and welfare and noted that it was one of their needs. The participants also highlighted their citizenship rights, the right of being accepted by society, and the right of enjoying all social facilities. In other words, they emphasized social welfare and acceptance. Leung et al. concluded that self-efficacy and adaptability of family members had a close relationship with the support received by the family (44). Several studies have found that satisfaction with life, the need for information about the treatment process, and available social support are the factors in the treatment and welfare of families (44). Marsack and Samuel argued that the responsibility of providing care to children with mental disorders had a negative effect on the quality of life of the parents. Informal social support and family support play an intermediating role between the responsibility of caring for a child with a disease and the quality of life of the parents (45). The results highlighted the need of providing support to the parents through informal social support. In developed countries, social and informal support for the patients' families is very strong; however, in the studied society, the support was lacking. This can be attributed to the culture of the society, people's views toward governmental support, and the position of non-governmental organizations (NGOs) in society.

### Strengths and limitations

By combining the qualitative and quantitative results, it is clear that there is a major overlap between the qualitative and quantitative results. In some cases, the qualitative results clearly elaborate on the families' needs in areas such as emotional support, the need for therapeutic and financial support, the need for the observation of their citizenship rights, and the need for being accepted by family, relatives, and society. In the quantitative phase and based on the questionnaire, these areas of need were less highlighted. Therefore, using a qualitative and quantitative approach in this study was a big step toward unveiling the actual needs of families of a member with mental illness.

The reluctance of participants to participate in the qualitative phase or give consent to voice recording was the main problem faced during this study. To solve this, the authors ensured the participants that the whole interviews will remain confidential and the results will be published anonymously.

## Conclusion

The family members of patients with mental disorders had different needs in the area of perceived social support. These needs were more tangible among children with mentally ill parents and parents of children with mental disorders. The results showed that the families needed support in the areas of information and non-judgmental interactions with friends, families, and relatives. With regard to the community, compliance with citizenship rights and financial support and creating a sense of wellbeing and security were among the important needs of these families. Based on the results, it can be suggested that similar studies should be conducted quantitatively or qualitatively, or both in different societies given that cultures and customs can influence people's attitudes and experiences.

## Data availability statement

The original contributions presented in the study are included in the article/supplementary material, further inquiries can be directed to the corresponding author.

## Ethics statement

The studies involving human participants were reviewed and approved by Ethical Committee of the Kermanshah University of Medical Sciences (IR.KUMS.1398.150). The patients/participants provided their written informed consent to participate in this study. Written informed consent was obtained from the individual(s) for the publication of any potentially identifiable images or data included in this article.

## Author contributions

SA contributed to the study concept, study design, data collection, and manuscript preparation. AJ contributed to the study concept, study design, data analysis, manuscript preparation, and submitting the manuscript. RJ contributed to the study concept, study design, data analysis, and manuscript preparation. All authors have read and approved the manuscript.
